# Neo-functionalization in *Saccharomyces cerevisiae*: a novel Nrg1-Rtg3 chimeric transcriptional modulator is essential to maintain mitochondrial DNA integrity

**DOI:** 10.1098/rsos.231209

**Published:** 2023-11-01

**Authors:** Carlos Campero-Basaldua, James González, Janeth Alejandra García, Edgar Ramírez, Hugo Hernández, Beatriz Aguirre, Nayeli Torres-Ramírez, Dariel Márquez, Norma Silvia Sánchez, Nicolás Gómez-Hernández, Ana Lilia Torres-Machorro, Lina Riego-Ruiz, Claudio Scazzocchio, Alicia González

**Affiliations:** ^1^ Departamento de Bioquímica y Biología Estructural, Instituto de Fisiología Celular Universidad Nacional Autónoma de México, Ciudad de Mexi, México; ^2^ Departamento de Genética Molecular, Instituto de Fisiología Celular Universidad Nacional Autónoma de México, Ciudad de Mexi, México; ^3^ Laboratorio de Biología Molecular y Genómica, Departamento de Biología Celular, Facultad de Ciencias, Universidad Nacional Autónoma de México, Ciudad de Mexico, México; ^4^ Laboratorio de Microscopía Electrónica Departamento de Biología Celular, Facultad de Ciencias, Universidad Nacional Autónoma de México, Ciudad de Mexico, México; ^5^ Departamento de Biología, Facultad de Química, UNAM, México City, Universidad Nacional Autónoma de México, Ciudad de Mexico, México; ^6^ División de Biología Molecular, Instituto Potosino de Investigación Científica y Tecnológica (IPICYT), San Luis Potosí, SLP, México; ^7^ Laboratorio de Biología Celular, Departamento de Investigación en Fibrosis Pulmonar, Instituto Nacional de Enfermedades Respiratorias ‘Ismael Cosío Villegas', Tlalpan, Mexico; ^8^ Department of Life Sciences, Imperial College London, London SW7 2AZ, UK; ^9^ Université Paris-Saclay, CEA, CNRS, Institute for Integrative Biology of the Cell (I2BC), 91198 Gif-sur-Yvette, France

**Keywords:** transcriptional coregulators, respiratory metabolism, mitochondrial genes, neo-functionalization

## Abstract

In *Saccharomyces cerevisiae,* the transcriptional repressor Nrg1 (Negative Regulator of Glucose-repressed genes) and the β-Zip transcription factor Rtg3 (ReTroGrade regulation) mediate glucose repression and signalling from the mitochondria to the nucleus, respectively. Here, we show a novel function of these two proteins, in which alanine promotes the formation of a chimeric Nrg1/Rtg3 regulator that represses the *ALT2* gene (encoding an alanine transaminase paralog of unknown function). An *NRG1/NRG2* paralogous pair, resulting from a post-wide genome small-scale duplication event, is present in the *Saccharomyces* genus. Neo-functionalization of only one paralog resulted in the ability of Nrg1 to interact with Rtg3. Both *nrg1*Δ and *rtg3*Δ single mutant strains were unable to use ethanol and showed a typical petite (small) phenotype on glucose. Neither of the wild-type genes complemented the petite phenotype, suggesting irreversible mitochondrial DNA damage in these mutants. Neither *nrg1*Δ nor *rtg3*Δ mutant strains expressed genes encoded by any of the five polycistronic units transcribed from mitochondrial DNA in *S. cerevisiae*. This, and the direct measurement of the mitochondrial DNA gene complement, confirmed that irreversible damage of the mitochondrial DNA occurred in both mutant strains, which is consistent with the essential role of the chimeric Nrg1/Rtg3 regulator in mitochondrial DNA maintenance.

## 1. Introduction

Gene duplication is a major evolutionary mechanism that provides raw material for generating genes with novel or modified physiological roles [1,2]. Phylogenomic studies have shown that in the ancestry of a clade of Saccharomycotina, including *Saccharomyces cerevisiae* and other related yeasts (post-whole genome duplication species, WGD), the occurrence of an interspecies hybridization event resulted in genome duplication [3,4]. Selective retention and sub-functionalization of gene pairs derived from the hybrid ancestor have led to the functional divergence of two paralogous copies [5–8] or to the neo-functionalization of one of the copies. The pairs of paralogous genes that are extant in *S. cerevisiae* could have originated from one of the parental species (ohnologous pairs), or each of the parental strains could have independently generated one member of the pair (homeologous genes). However, duplication could have arisen independently preceding or following the WGD event. Duplication and neo-functionalization have been described for genes involved in primary metabolism such as amino acid biosynthesis [8–10] and genes encoding transcription factors [11–14].

*ALT1* and *ALT2* encode proteins with 65% identity and are assumed to be paralogous alanine transaminases [15]. Additional phylogenetic studies suggested that *ALT1* and *ALT2* originated in one of the two distinct parental strains, which gave rise to the ancestral hybrid [16]. *ALT1* encodes an alanine transaminase involved in both the main alanine biosynthetic pathway and in the sole alanine catabolic pathway [17]. *ALT2*, despite its sequence similarity with *ALT1* (65% identity), is not an alanine transaminase and has no role in alanine metabolism; in fact, its physiological function remains unknown [9,10]. Alt1 and Alt2 subcellular localization has also diverged since Alt1 is localized in the mitochondrial matrix while Alt2 is cytosolic [18]. Most interesting was the finding that Alt1 has been considered to be a moonlighting protein since, besides its role in alanine catabolism, it contributes to the maintenance of mitochondrial DNA integrity and mitochondrial gene expression [19].

In addition to its role in protein biosynthesis, alanine plays a signaling role as a co-activator or co-repressor; accordingly, *ALT1* expression is induced by alanine [17], while that of *ALT2* is repressed by alanine. Analysis of *ALT1* and *ALT2* gene promoters showed that both promoters carry an Nrg1 binding consensus sequence, while *ALT1* additionally includes an Rtg3 binding sequence. The present study shows that although the *ALT2* gene promoter does not include an Rtg3 binding sequence, both Nrg1 and Rtg3 are necessary for alanine-dependent *ALT2* repression, which is alleviated in both *nrg1*Δ and *rtg3*Δ single mutants. These observations suggested the formation of a previously unreported Nrg1-Rtg3 chimeric complex. The roles of Nrg1 [20] and Rtg3 [21] as transcriptional regulators have been previously identified and thoroughly analysed. Nrg1 and its paralog Nrg2, functioning as transcriptional repressors, regulate a group of stress-responsive genes [20,22]. The first group of genes, which were found to be repressed by Nrg1/Nrg2 belonged to the glucose-repressed circuit. Further studies have shown that these two paralogs are also involved in responses to other environmental stresses, such as alkaline pH stress response, hyperosmotic salinity response, negative regulation of pseudohyphal growth, and biofilm formation [23]. The *NRG1*/*NRG2* pair arose as a post-WGD small-scale duplication event that occurred within the *Saccharomyces* genus, *NRG2* being the ancestral gene present in other Saccharomycotina (data in PhylomeDB 5 http://phylomedb.org/ [16]).

Rtg3, together with Rtg1 (not a paralog of Rtg3), forms a complex that activates the retrograde signalling pathway from the mitochondria to the nucleus, promoting the transcriptional activation of several genes [21]. Activation of the retrograde response extends the yeast replicative lifespan, which is enhanced by the progressive decline in mitochondrial membrane potential during ageing [24,25]. Here, we demonstrate that *ALT2* alanine-dependent repression necessitates the presence of the Nrg1-Rgt3 chimeric regulator, implying a novel physiological role for the Nrg1-Rtg3 chimera, which is not present in either Nrg1 or Rtg3. The novel regulatory role played by chimeric modulators has previously been found for the Hap2-3-5-Gln3 and the Gln3-Gcn4 hybrid chimeras, comprising the Hap2-3-5-4 and Gln3 regulators and Gln3 and Gcn4, respectively [26,27].

Forsburg and Guarente [28] proposed the possible existence of chimeric regulators when they established Hap4 as a member of the HAP complex [28]. They argued that, since the HAP complex is composed of a DNA-binding domain involving three polypeptides (Hap2-3-5) and an independent activator sequence included in the Hap4 protein, the combination of these peptides could generate novel chimeric regulators. The Hap2-3-5-Gln3 hybrid regulator, which allows Gln3-dependent *GDH1* transcriptional activation even in the presence of repressive nitrogen sources such as glutamine, is a striking example [27], which confirms the prediction of Forsburg & Guarente [28]. In all cells, multiple DNA-binding proteins play crucial roles in the specificity of transcriptional responses; the existence of chimeric transcriptional modulators increases the repertoire of regulatory possibilities [26–28]. Recent studies on the formation of cooperative protein–protein interactions between two ancient transcriptional regulators (Mat*a*2 and Mcm1) in ascomycetes yeasts, including *Saccharomyces cerevisiae*, have highlighted that the formation of novel and alternative combinations of regulators over evolutionary time scales represents a major source of phenotypic novelty, which could promote a novel network organization [29].

Immunoprecipitation results included in this study demonstrated the formation of an Nrg1/Rtg3 transcriptional chimera, whose DNA-binding domain is that of Nrg1, while both Rtg3 and Nrg1 are involved in chromatin organization of the *ALT2* promoter.

Our study of this chimeric regulator led to unexpected results. The Nrg1/Rtg3 regulator plays a far more crucial role than the alanine-mediated repression of *ALT2*. This is essential for the maintenance of mitochondrial DNA integrity. The absence of either component resulted in gross, irreversible and altered mitochondrial DNA. Formation of the Nrg1-Rgt3 complex implies neo-functionalization of Nrg1*,* as the Nrg2 paralog cannot function in the maintenance of mitochondrial DNA integrity. We argue that this neo-functionalization is derived from the newly acquired ability to form a chimeric complex with Rtg3.

## 2. Methods

### 2.1. Strains

Table 1 and electronic supplementary material, table S1 describe the characteristics of the strains and plasmids used in this study. The CLA1 *ura3 leu2* construction was previously described [30]. The CLA1-2 (*ura3 leu2*::*LEU2*) [6] strain was transformed with plasmid pRS416 obtaining the CLA1-2-A*/*pRS416 strain used as a control harbouring the empty plasmid. The construction of strain CLA11-713 (*nrg1*Δ::*kanMX4 ura3 leu2*::*LEU2*) has been described previously [8]. CLA11-714 (*nrg2*Δ::*kanMX4 ura3*Δ *leu2*Δ) was obtained via gene replacement. A polymerase chain reaction (PCR)-generated *kanMX4* module was prepared from the plasmid pFA6a (electronic supplementary material, table S1) using R5 and R6 deoxyoligonucleotides (electronic supplementary material, table S2a). The CLA11-715 double mutant (*nrg1*Δ::*natMX4*; *nrg2*Δ:: *kanMX4 ura3*Δ *leu2*Δ) was constructed as follows. The *kanMX4* module from (*nrg1*Δ::*kanMX4 NRG2 ura3 leu2*) was replaced by the *natMX4* cassette, which confers resistance to the nourseothricin antibiotic [31]. The *natMX4* module used for transformation was obtained from the plasmid p4339 (electronic supplementary material, table S1) using R3 and R4 deoxyoligonucleotides (electronic supplementary material, table S2a). The module obtained for *nrg1*Δ::*natMX4* was transformed into the *nrg2*Δ::*kanMX4* strain. A CLA1-2 isogenic CLA1-3 (*rtg3*Δ) derivative was obtained by gene replacement. One PCR-generated *kanMX4* module prepared from plasmid pFA6a (electronic supplementary material, table S1) and deoxyoligonucleotides R1–R2 (electronic supplementary material, table S2a) was used to generate the corresponding *RTG3* module, following a previously described method [32]. Strain CLA1-2 was transformed with the 2612 bp PCR product containing the *kanMX4* cassette and *RTG3* upstream or downstream nucleotide sequences amplified from the genomic DNA of the CLA1-2 strain (electronic supplementary material, table S2a).

**Table 1.** Strains used in the present work.

strain	relevant genotype	source
CLA1	*MATα ALT1 ALT2 NRG1 RTG3 ura3 leu2*	Valenzuela *et al*. [30]
CLA1-2	*MATαALT1 ALT2 NRG1 RTG3 ura3 leu2::LEU2*	Quezada *et al.* [6]
CLA1*/*pRS416	*MATα ALT1 ALT2 NRG1 RTG3/*pRS416 *leu2*	this study
CLA1-2*/*pRS416	*MATα ALT1 ALT2 NRG1 RTG3/*pRS416 *leu2::LEU2*	this study
CLA1-2-1(*alt1*Δ)	*MATα alt1*Δ*::kanMX4 ALT2 NRG1 RTG3 ura3 leu2::LEU2*	García-Campusano *et al.* [17]
CLA1-2-1(*alt1*Δ /pRS416)	*MATα alt1*Δ*::kanMX4/*pRS416 *ALT2 NRG1 RTG3 leu2::LEU2*	this study
CLA1-2-1(*alt1*Δ /pRS416*-ALT1*)	*MATα alt1*Δ*::kanMX4/*pRS416*-ALT1 ALT2 NRG1 RTG3 leu2::LEU2*	this study
CLA11-713 (*nrg1*Δ)	*MATα ALT1 ALT2 nrg1*Δ*::kanMX4 RTG3 ura3 leu2*	González *et al.,* [8]
CLA11-714 (*nrg2*Δ)	*MATα ALT1 ALT2 nrg2*Δ*::kanMX4 NRG1 RTG3 ura3 leu2*	this study
CLA11-715 (*nrg1*Δ *nrg2*Δ)	*MATα ALT1 ALT2 nrg1*Δ*::natMX4, nrg2*Δ*::kanMX4 RTG3 ura3 leu2*	this study
CLA11-713 (*nrg1*Δ*/*pRS416)	*MATα ALT1 ALT2 nrg1*Δ*::kanMX4/*pRS416 *RTG3 leu2*	this study
CLA11-713 (*nrg1*Δ*/*pRS416*-NRG1*)	*MATα ALT1 ALT2 nrg1*Δ*::kanMX4/*pRS416*-NRG1 RTG3 leu2*	this study
CLA1-3 (*rtg3*Δ)	*MATα ALT1 ALT2 NRG1 rtg3*Δ*::kanMX4 ura3 leu2*	this study
CLA1-3 (*rtg3*Δ*/*pRS416)	*MATα ALT1 ALT2 NRG1 rtg3*Δ*::kanMX4*/pRS416 *leu2*	this study
CLA1-3 (*rtg3*Δ*/*pRS416*-RTG3*)	*MATα ALT1 ALT2 NRG1 rtg3*Δ*::kanMX4*/pRS416*-RTG3 leu2*	this study
CLA1-604 (*NRG1^-Myc13^*)	*MATα ALT1 ALT2 NRG1*^-^*^Myc13^::kanMX4 RTG3 ura3 leu2*	Peñalosa-Ruiz *et al*., [33]
CLA1-605 (*RTG3^-Myc13^*)	*MATα ALT1 ALT2 NRG1 RTG3*^-^*^Myc13^::kanMX4 ura3 leu2*	this study
BY4741 (*RTG3^-TAP^*)	*MATa ALT1 ALT2 NRG1 RTG3^-TAP^::HIS3 ura3 leu2 met15*	Open Biosystems
BY4741 (*RTG3^-TAP^-NRG1^-Myc13^*)	*MATa ALT1 ALT2 NRG1*^-^*^Myc13^::kanMX4 RTG3^-TAP^::HIS3 ura3 leu2 met15*	this study
BY4742	*MATα ALT1 ALT2 NRG1 RTG3 ura3 leu2 his3 lys2*	Open Biosystems
BY4742 (*nrg1*Δ)	*MATα ALT1 ALT2 nrg1*Δ*::kanMX4 RTG3 ura3 leu2 his3 lys2*	Open Biosystems
BY4742 (*rtg3*Δ)	*MATα ALT1 ALT2 NRG1 rtg3*Δ*::kanMX4 ura3 leu2 his3 lys2*	Open Biosystems
BY4742 (rho0)	*MATα ALT1 ALT2 NRG1 RTG3 ura3 leu2 his3 lys2 р^0^*	provided by Xóchitl Pérez-Martínez and Yolanda Camacho-Villasana

### 2.2. Construction of Myc13-tagged strains

An *RTG3*-^TAP^ mutant was obtained from the BY4741 TAP-tagged *Saccharomyces* strain (BY4741 *ura3 leu2 his3 met5 RTG3*^-^*^TAP^*::*HIS3*). The BY4741 *RTG3^-^^TAP^* *NRG1^-^^Myc13^* derivative was obtained as previously described [32]. A pair of deoxyoligonucleotides, T1 and T2 (electronic supplementary material, table S2b), was designed based on the *NRG1* coding sequence and that of the pFA6a-13Myc-*kanMX6* multiple cloning site [32], generating a PCR-13Myc*-kanMX6* module of 2300 bp, which was used to transform the BY4741 *RTG3*^-^*^TAP^* tagged strain, generating the mutant strain *RTG3*^-^*^TAP^* *NRG1*^-^*^Myc13^* double-tagged derivative. Deoxyoligonucleotides T3 and T4 (electronic supplementary material, table S2*b*) were used to verify the construction *RTG3*^-*TAP*^ *NRG1*^-*Myc13*^*.* These primers generated a 2673 bp module (233 bp of *NRG1* + 2300 bp of 13Myc*-kanMX6* + 137 bp of the 3'UTR of *NRG1*). Construction of the *NRG1*^-^*^Myc13^* single-strain mutant has been previously described [33]. An *RTG3*^-*Myc13*^ single-strain mutant was constructed as follows. A pair of deoxyoligonucleotides, T5 and T6 (electronic supplementary material, table S2b), was designed based on the *RTG3* coding sequence and that of the pFA6a-13Myc-*kanMX6* multiple cloning site [32], generating a PCR-13Myc*-kanMX6* module of 2300 bp, which was used to transform the *CLA1 RTG3 NRG1* to obtain the single *RTG3*^-^*^Myc13^* strain. In order to confirm the correct insertion of *RTG3*^-^*^Myc13^* we used T7 and T8 oligonucleotides (electronic supplementary material, table S2b) to amplify the 2874 bp module.

### 2.3. Growth conditions

Strains were routinely grown on minimal medium (MM) containing salts, trace elements and vitamins, according to the formula for yeast nitrogen base (Difco). Glucose (2% w/v) or ethanol (2% v/v) was used as the carbon source; 7 mM Gamma-aminobutyric acid (GABA), 8 mM proline, 7 mM alanine, or 40 mM ammonium sulfate were used as nitrogen sources. Uracil (20 mg l^−1^), 7 mM leucine or glutamate (20 mg l^−1^) were added as auxotrophic requirements when needed. Cells were incubated at 30°C with shaking (250 r.p.m.).

### 2.4. *NRG1* and *RTG3* cloning

*ALT1* cloning was performed as previously described [10]. For *NRG1* cloning, two variants of the *NRG1*-containing module were created, and the AB and CD constructs (deoxyoligonucleotides are listed in electronic supplementary material, table S2c). The AB region consists of a 2492 bp PCR product (deoxyoligonucleotides CN1 and CN2) harbouring −1512 nucleotides from the *NRG1* start codon and +284 nucleotides from the *NRG1* stop codon. The CD region consists of the 2902 bp PCR product (oligonucleotides CN3 and CN4) harbouring −1867 nucleotides from the *NRG1* start codon and +339 nucleotides from the *NRG1* stop codon. Recognition sites for the endonuclease restriction enzymes *BamHI* and *XhoI* were added to the forward and reverse deoxyoligonucleotides, *NRG1* PCR products, and the pRS416 (*CEN6 ARS4 URA3*) plasmid (electronic supplementary material, table S1) were *BamHI*-*XhoI* digested and ligated after gel purification. Ligations were transformed into a *DH5α Escherichia coli* strain. Plasmids were purified from *E. coli* extracts, and after correct cloning was verified by sequencing, the plasmids were transformed into *S. cerevisiae* strains CLA1 *NRG1* and CLA11-713 *nrg1*Δ. Transformants were selected for uracil prototrophy in MM).

For *RTG3* cloning, a 2769 bp PCR product using deoxyoligonucleotides CR1 and CR2 (electronic supplementary material, table S2c), harbouring −1089 nucleotides from the start codon and +219 nucleotides from the *RTG3* stop codon. Recognition sites for endonuclease restriction enzymes *BamHI* and *XhoI* were added to forward and reverse deoxyoligonucleotides, respectively. *RTG3* PCR product and pRS416 (*CEN6 ARS4 URA3*) plasmid (electronic supplementary material, table S2) were *BamHI*-*XhoI* ligated after gel purification. Ligations were transformed into *DH5α Escherichia coli* strain. Plasmids were purified, and cloning was verified by sequencing. The construct cloned in pRS416 was transformed into *S. cerevisiae* strains CLA1 *RTG3* and CLA1-3 *rtg3*Δ. Transformants were selected for uracil and glutamic acid prototrophy in MM.

### 2.5. Northern blot analysis

Northern blot analysis was performed as previously described [8]. Total yeast RNA was prepared from 200 ml cultures grown to the indicated OD_600nm_. Probes to monitor the expression of *ALT1, ALT2, CIT2*, *HXT2* and *ACT1* were prepared by PCR from CLA1-2 genomic DNA using primers N1 to N10 (electronic supplementary material, table S2*d*) and radioactively labelled P^32^ with Random Primer Labeling kit (Agilent, 300385). Blots were scanned using the Image Quant 5.2 software (Molecular Dynamics).

### 2.6. Quantitative polymerase chain reaction differential transcript expression analysis

*Saccharomyces cerevisiae* strain was grown on MM glucose (2% w/v) as a carbon source and supplemented with GABA (7 mM) or GABA + Ala (7 mM) as nitrogen sources to an OD_600nm_ of 0.3. The cells were harvested by centrifugation at 3000 r.p.m. for 5 min and washed twice with distilled H_2_O. The cells were then transferred to a mortar, frozen with liquid nitrogen and mechanically disrupted with the aid of a pestle. Total RNA was extracted using TRIzol reagent (Invitrogen) following the TDS procedure. The RNA quality and concentration were determined using a NanoDrop 2000 spectrophotometer (NanoDrop Technologies). RNA was treated with RQ1 RNAse-Free DNAse (Promega) to degrade DNA from the samples; afterwards, cDNA was obtained using the RevertAid H Minus First Strand cDNA Synthesis Kit, following the manufacturer's instructions (Thermo Scientific). cDNA samples were adjusted to a concentration of 30 ng µl^−1^. PCR was performed using the KAPA SYBR Fast kit (Roche) and run on the Corbett Research Rotor-Gene 6000 (Qiagen). Gene expression was obtained for *ALT2*, *COX2*, *COX3*, *COB1*, *ATP9*, *ATP6* and *COX8* using deoxyoligonucleotides P1 to P14 (electronic supplementary material, table S2e). Data were analysed using the 2^^−ΔΔCT^ method, in which the RDN18S (18S) constitutive gene was used as a housekeeping control [34]. Statistical analysis was performed using GraphPad Prism 9.0 (GraphPad Software Inc). All experiments were repeated three times, and the results are expressed as mean ± s.e.m.

### 2.7. Nucleosome scanning assay

The nucleosome scanning assay (NuSA) was performed as previously described [35]. Cultures (100 ml) containing glucose (2% w/v) as a carbon source and supplemented with GABA (7 mM) or GABA + Ala (7 mM) as nitrogen sources were grown at OD_600nm_ = 0.3. To determine the nucleosome position in *ALT2* promoter, samples were treated as previously described [8,36]. Quantitative PCR (qPCR) analysis was performed using a Corbett Life Science Rotor Gene 6000 and SYBR Green dye (2X KAPA SYBR FAST, Invitrogen). Real-time PCR was performed as follows: 94°C for 5 min (1 cycle), 94°C for 15 s, 58°C for 20 s, and 72°C for 20 s (35 cycles). The PCR deoxyoligonucleotides (B1 to B23) used for *ALT2* analysis are described in electronic supplementary material, table S2g. Relative protection was calculated as a ratio to the control of *VCX1*, which was amplified using P19 and P20 oligonucleotides (electronic supplementary material, table S2e).

### 2.8. Immunoprecipitation of Rtg3^-^*^TAP^* and Nrg1^-^*^Myc13^*

This method was performed using a modified version of Gerace & Moazed [37]. *Saccharomyces cerevisiae* strains were grown on MM) with glucose (2% w/v) as a carbon source supplemented with *γ*-aminobutyric acid (GABA) 7 mM, GABA + alanine 7 mM (figure 2*a,b*), GABA 7 mM or proline 8 mM + increasing alanine concentrations (figure 2*c*), and harvested at OD_600nm_ = 0.5 by centrifugation at 3000 r.p.m. for 5 min, and washed with TBS 1X. Later, microtubes (2 ml) with the pellets were frozen, submerging them in liquid nitrogen for 15 s. 500 µl of lysis buffer (HEPES 50 mM, NaOAc 200 mM, EDTA 0.1 mM, EGTA 0.1 mM, MgOAc 5 mM, glycerol 5%, NP-40 0.25%, DTT 3 mM, PMSF 1 mM, protease inhibitor cocktail) and 400 µl of 0.5 mm glass beads were added and vortexed for 4 min twice with a pause of 2 min on ice. The microtube was centrifuged at 14 000 r.p.m. for 5 min at 4°C, the total protein lysate was transferred to a new 2 ml microtube. Protein concentration was measured using the Bradford method. The normalized protein concentration (2000 µg or 4000 µg) was added to a microtube containing 60 µl of Pierce protein A/G agarose beads (Thermo Scientific) and 10 µl of TAP antibody CAB1001 (Invitrogen), 10 µl of Myc13 antibody 9E11 (Santa Cruz Biotechnology), or 10 µl of HA antibody sc-7392 (Santa Cruz Biotechnology). Samples were incubated with mild agitation for 2 h at 4°C. After, samples were washed five times with 1 ml of wash buffer (HEPES 50 mM, NaOAc 200 mM, EDTA 0.1 mM, EGTA 0.1 mM, MgOAc 5 mM, glycerol 5%, NP-40 0.25%, DTT 3 mM, PMSF 1 mM) centrifuged at 2000 r.p.m. for 1 min at 4°C. Subsequently, all supernatants were removed and 50 µl of loading buffer (Tris base 50 mM pH 6.8, SDS 2%, glycerol 10%, DTT 100 mM, PMSF 1 mM, bromophenol blue 0.001%) was added and incubated at 65°C for 10 min. Forty microlitres were run on 10% SDS-PAGE for Western blot analysis using anti-TAP antibodies (Thermo Scientific).

### 2.9. Quantitative chromatin immunoprecipitation

Formaldehyde cross-linking and immunoprecipitation were performed according to a previously described procedure [27]. Yeast cells (200 ml of OD_600nm_ = 0.3) were cross-linked with 1% formaldehyde for 20 min at room temperature. Afterwards, 125 mM glycine was added, and the mixture was incubated for 5 min. The cells were harvested and washed with PBS. The pelleted cells were suspended in lysis buffer (140 mM NaCl, 1 mM EDTA, 50 mM HEPES/KOH, 1% Triton X-100 and 0.1% sodium deoxycholate) with a protease inhibitor cocktail (Complete Mini, Roche). The cells were lysed with glass beads and collected by centrifugation. Extracts were sonicated using a Diagenode Bioruptor to produce chromatin fragments with an average size of 300 bp. Immunoprecipitation reactions were carried out with 1 mg anti-c-Myc antibody (9E11, Santa Cruz Biotechnology) and protein A beads for 3 h, washed, suspended in TE buffer/1% SDS, and incubated overnight at 65°C to reverse the formaldehyde cross-linking. Immunoprecipitants were then incubated with proteinase K (Roche), followed by phenol/chloroform/isoamyl alcohol extraction, precipitation and suspension in 30 µl of TE buffer. Dilutions of input DNA (1 : 100) and immunoprecipitated DNA (1 : 2) were analysed by qPCR. Real-time PCR-based DNA amplification was performed using primers that were initially screened for dimer absence or cross-hybridization. Only the primer pairs (C1 to C18) with similar amplification efficiencies were used (electronic supplementary material, table S2f). Quantitative chromatin immunoprecipitation (qChIP) analysis was performed using a Corbett Life Science Rotor Gene 6000 instrument. The fold difference between immunoprecipitated material (IP) and the total input sample for each qPCR-amplified region was calculated using the formula IP/input = (2^^IPCt^/2^^InputCt^) [38]. The results represent the mean values and s.e. of at least three independent cross-linked samples, each immunoprecipitated twice with the antibody.

### 2.10. Ethanol measurement

For ethanol determination, cells were grown to the indicated OD_600nm_ in glucose and ammonium sulfate as nitrogen sources. Aliquots (1 ml) were withdrawn for supernatant recovery by centrifugation at 14 000 r.p.m. Ethanol was quantified in the supernatant following a previously reported protocol [39] using a reaction buffer (bicine-KOH 20 mM, pH 9.0, 1 U ml^−1^ alcohol dehydrogenase, Sigma Aldrich A7011). The assay was carried out at 340 nm, 25°C in a Variant Cary 50 spectrophotometer. A standard curve of ethanol to calculate sample concentration was used [19].

### 2.11. Fluorescent microscopy

Cells were stained with Green MitoTracker FM 9074 (Molecular Probes), according to the manufacturer's specifications. Confocal images were obtained using a FluoView FV1000 laser confocal system (Olympus) attached/interfaced to an Olympus IX81 inverted light microscope with a 60x oil-immersion objective (UPLASAPO 60x O NA : 1.35), zoom x 20.0 and 3.5 µm of confocal aperture. The excitation and emission settings were as follows: MitoTracker excitation, 543 nm; emission, 598 nm; and BF, 555 nm range 100 nm. Subsequent image processing was performed using Olympus Fluo View FV1000 (v. 1.7) software.

### 2.12. Quantification of mtDNA/nDNA ratio

To quantify the mtDNA we obtained the amount of mtDNA relative to nDNA. We followed the method described by [19], estimating the mtDNA/nDNA ratio by qPCR using different genes of mtDNA: *COX2*, *COX3* and *ATP6*, and as a nuclear-encoded gene, we selected *COX8*. Total DNA from the indicated strains was extracted twice by phenol/chloroform with 20 µl of NaCl 5 M. Samples were incubated with 20 µg RNase A for 1 h at 37°C. DNA precipitation was carried out with an equal volume of ethanol for 30 min at −20°C and resuspended in nuclease-free water. DNA was quantified by NanoDrop (Thermo Scientific), and samples were diluted to 50 ng µl^−1^ for qPCR assay. qPCR was performed using SYBR Green dye (2X KAPA SYBR FAST; Invitrogen). conditions for qPCR were 94°C for 5 min (one cycle), 94°C for 15 s, 59°C for 30 s, and 72°C for 20 s (30 cycles). We obtained the cycle threshold (Ct) for each sample, and following the 2^^ΔΔCt^ method, we calculated the mtDNA/nDNA ratio as described previously [40].

### 2.13. Phylogeny of yeast proteins

The PhylomeDB (http://phylomedb.org/) database was used [41] to analyse Nrg1 and Nrg2 phylogeny.

### 2.14. Structural modelling and visualization

The extant structural models were downloaded from the AlphaFold database (https://alphafold.ebi.ac.uk/) [41]. A chimeric complex model was obtained using AlphaFold2.ipynb (https://colab.research.google.com/github/sokrypton/ColabFold/blob/main/AlphaFold2.ipynb#scrollTo=ADDuaolKmjGW) [42].

The contacts between chains were determined using the MAPIYA contact map server (https://mapiya.lcbio.pl/) [43]. Structural models were visualized, and figures were drawn with ChimeraX-1.5 for Nrg1-Nrg2 chain comparison and with VMD 1.9.4 (http://www.ks.uiuc.edu/Research/vmd/) for visualization of the Nrg1-Rtg3 complex and inter-chain contacts [37,44,45].

## 3. Results

### 3.1. Nrg1/Rtg3 forms a chimeric transcriptional complex whose assembly is promoted by alanine

**Figure 1.**
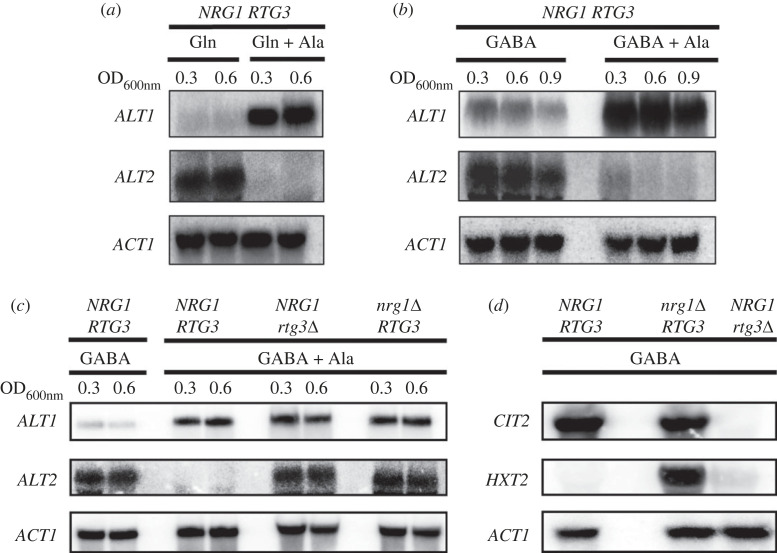
Alanine induces *ALT1* and represses *ALT2*. For Northern blot analysis, cultures were grown at 30°C on glutamine (7 mM) and GABA (7 mM) with or without alanine (7 mM). (*a*) glutamine (Gln) and glutamine + alanine (Gln + Ala), (*b*) γ-aminobutiric acid (GABA) and GABA + alanine (GABA + Ala) and (*c*) GABA (7 mM) and GABA + alanine (GABA + Ala). When each culture reached an OD_600nm_ = 0.3, samples from each culture were taken for RNA extraction. A second sample of each culture was taken when cells reached an OD_600nm_ = 0.6, and RNA extraction was performed. Finally, a third sample was taken from (*b*) cultures when these reached 0.9 OD_600nm_. (*d*) Cultures were grown on GABA to OD_600nm_ = 0.3 and expression of *CIT2* (citrate synthase) was monitored as a control of Rtg3 activity as a positive regulator, and *HXT2* (glucose transporter) as a target of Nrg1 as a repressor. All samples were prepared for RNA extraction followed by Northern blot as described in Methods.

As mentioned earlier, *ALT1* and *ALT2* arose from interspecies hybridization, resulting in a WGD event [4,46]. Alanine-dependent *ALT1* induction and *ALT2* repression were observed when cells were grown in the presence of alanine either on repressive nitrogen sources such as glutamine or on non-repressive nitrogen sources such as GABA (figure 1*a,b*).

Analysis of the *ALT1* and *ALT2* gene promoter sequences showed that both promoters harbour DNA consensus-binding sites for Nrg1 and that *ALT1* additionally bears an Rtg3 DNA consensus-binding site. To analyse whether Nrg1 and/or Rtg3 play a role in *ALT1* and *ALT2* expression, null *NRG1* and *RTG3* mutants were constructed. Northern blot analysis showed (figure 1*c*) that both *nrg1*Δ and *rtg3*Δ mutants retained *ALT1* alanine-dependent induction, while *ALT2* repression did not occur in either mutant. We confirmed the previously described independent roles of *NRG1* and *RTG3* by monitoring the expression of *CIT2* (citrate synthase) and *HXT2* (high-affinity glucose transporter) in each deletion mutant strain. As expected, *CIT2* expression was solely dependent on Rtg3 positive regulation (figure 1*d*) [21], whereas that of *HXT2* was regulated by Nrg1-dependent repression (figure 1*d*) [47].

**Figure 2.**
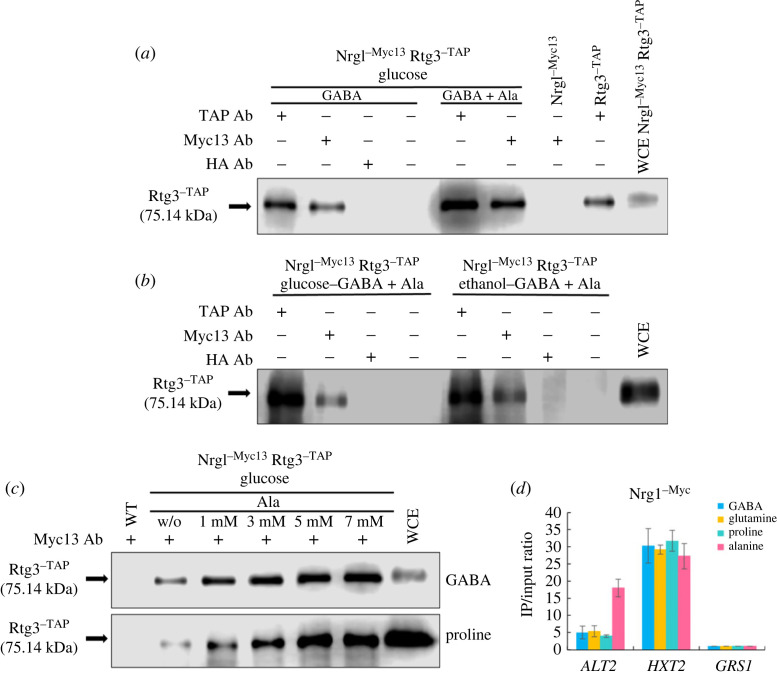
Rtg3 and Nrg1 form a transcriptional complex. Total protein samples were obtained from the double-tagged Nrg1^-^*^Myc-13^* Rtg3^-^*^TAP^* strain, and single-tagged Nrg1^-^*^Myc-13^* or Rtg3^-^*^TAP^* strains and processed as described in Methods. Co-immunoprecipitation (CoIP): (*a*) compares the relative quantities of Rtg^-^*^TAP^* coprecipitated in the presence or absence of alanine (7 mM). Extracts of the double mutant carrying gene fusions of the cognate genes with Myc-13 or TAP (see Methods) Nrg1^-^*^Myc13^* Rtg3^-^*^TAP^* were used for the immuno- and co-immunoprecipitation tests in the first six lanes. Single Nrg1^-^*^Myc-13^* and Rtg3^-^*^TAP^* tagged mutants grown on GABA (7 mM) + alanine (7 mM) were used as controls for Western blots revealed with anti-TAP. As a control, a whole cell extract (WCE) of the double-tagged strain grown on YPD was used to determine Rtg3^-^*^TAP^* localization in the gel. Mouse haemagglutinin antibody (HA-Ab) was used as a negative control, and the fourth lane did not carry antibodies. (*b*) Rtg3^-^*^TAP^* and Nrg1^-^*^Myc-13^* co-immunoprecipitation of extracts obtained from cultures grown on either MM 2% glucose or MM 2% ethanol. (*c*) Dependence of Rtg3 coprecipitated with Nrg1 in extracts obtained from yeast cells grown on GABA (7 mM) or proline (8 mM), in the alternative presence of increasing alanine concentrations in the medium. Immunoprecipitation tests were carried out separately with anti-Myc-13 (9E11 Santa Cruz Biotechnology), anti-TAP (Thermo Scientific) and anti-HA (F-7 Santa Cruz Biotechnology) as indicated. Samples (30 µl) were run on a 10% SDS-PAGE gel, and Western blot analysis was carried out with anti-TAP antibody. A 6 µl sample of WCE was used as a Rtg3-TAP control. (*d*) qChip assays were carried out on *ALT2*, *HXT2* and *GRS1* gene promoters in extracts obtained from the Nrg1^-^*^Myc-13^* tagged strain grown on MM in the presence of either 7 mM GABA, glutamine, proline or alanine. *HXT2* (glucose transporter) was used as positive control and *GRS1* (glycyl-tRNA synthase) as negative control.

*ALT2-* Nrg1- and Rtg3-dependent repression suggested that these regulators might form an Rtg3-Nrg1 chimeric repressor. To analyse this possibility, single and double RTG3^-^*^TAP^* NRG1^-*Myc13*^ mutants were constructed, as described in the Methods section. As shown in figure 2*a*, in a cell extract prepared from an *RTG3^-^^TAP^* *NRG1*^-*Myc13*^ double-tagged strain immunoprecipitated with an anti-Myc13 antibody, the presence of Rtg3^-*TAP*^ was revealed after Western blot analysis with an anti-TAP antibody, indicating that Rtg3 and Nrg1 are present in the same complex. Co-immunoprecipitation was performed with either glucose or ethanol as the carbon source (figure 2*b*). Interestingly, co-immunoprecipitation enrichment was observed when the cells were grown in the presence of alanine (figure 2*a,b*). To further analyse this observation, experiments were carried out using increasing alanine concentrations. As expected, alanine-dependent co-immunoprecipitation was proportionally higher in extracts obtained from cultures grown on 5 mM alanine than in those grown on 1 mM alanine (figure 2*c*). These results suggest that alanine promotes the formation of the Nrg1–Rtg3 chimeric complex. However, Nrg1^-^*^Myc13^* and Rtg3^-^*^TAP^* co-immunoprecipitation was also observed in extracts prepared from cultures grown on GABA or proline in the absence of externally added alanine (figure 2*c*). This may be promoted by the endogenous alanine pool (see Discussion section), suggesting that either the complex can be assembled without alanine or that the endogenously biosynthesized alanine pool can promote Nrg1–Rtg3 chimeric complex formation. Nrg1-^Myc13^ promoter occupancy was analysed using chromatin immunoprecipitation (ChIP) experiments. Immunoprecipitants were obtained from the Nrg1^-*Myc13*^ epitope-tagged strains, an anti-Myc antibody. Amplification of *HXT2* and *GRS1* coding sequences were used as controls. As shown in figure 2*d*, in the presence of alanine in the growth medium, Nrg1^-^*^Myc13^* chromatin immunoprecipitation was increased 10-fold as compared with that observed in the *ALT2* promoter in the absence of alanine. This contrasts with the results for the *HXT2* promoter, in which Nrg1^-*Myc13*^ was similarly recruited in the presence of 7 mM glutamine, proline, GABA, or alanine. These results indicate that since higher amounts of the Nrg1-Rtg3 complex are formed on an alanine medium, proportionally higher recruitment to the *ALT2* promoter must occur.

### 3.2. DNA binding is exclusively Nrg1-dependent, while Nrg1 and Rtg3 determine chromatin remodelling

**Figure 3.**
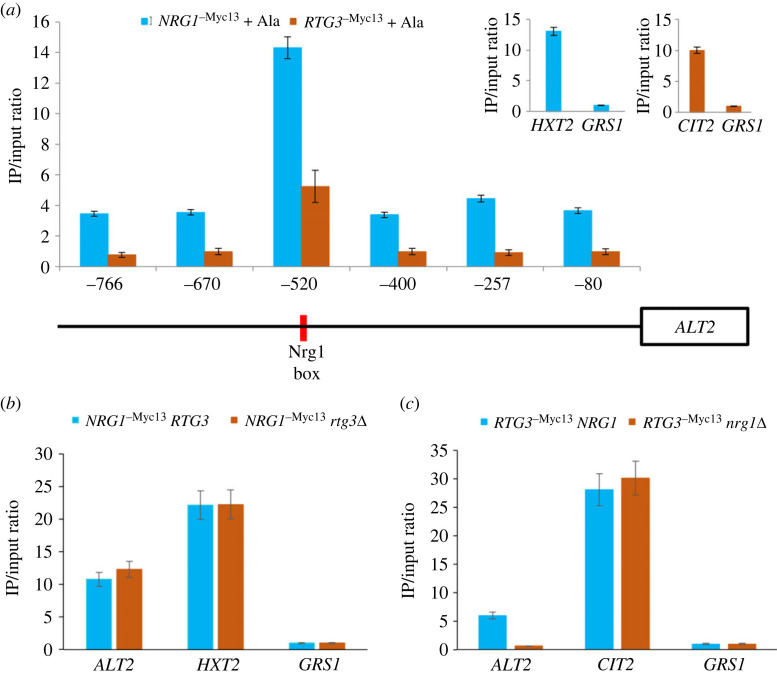
In the Nrg1–Rtg3 chimeric complex, the DNA binding domain is afforded by Nrg1. (*a*) qChip assays were carried out on the *ALT2* promoter region. The six DNA regions which were amplified after qChip are shown as vertical segments on the horizontal line representing various *ALT2* promoter regions. qChip assays were carried out with the anti-Myc-13 antibody (9E11, Santa Cruz Biotechnology) on WT strains containing Myc-13 epitope-tagged Nrg1^-^*^Myc-13^* or Rtg3^-^*^Myc-13^*. Strains were grown on MM glucose (2%) + GABA (7 mM) and binding was analysed by qChip as described in Methods. IP/input ratios were normalized with the *GRS1* promoter as negative control. *HTX2* (glucose transporter) and *CIT2* (citrate synthase) promoters were used as positive controls (shown in the right-side upper panels). In the *ALT2* depicted promoter, the vertical red line shows Nrg1 consensus binding sequence. (*b*) qChip assays were carried out on the *NRG1^-^^Myc-13^* *RTG3* and *NRG1^-^^Myc-13^* *rtg3*Δ. (*c*) qChip assay was carried out for the *RTG3*^-^^Myc-13^ *NRG1* and *RTG3*^-^^Myc-13^ *nrg1*Δ. For (*b*) and (*c*) *HXT2* and *CIT2* promoters were respectively used as positive controls, IP/input ratios were normalized with the *GRS1* promoter as the negative control. Data are presented as the average of three independent experiments.

To analyse whether the Nrg1 DNA consensus-binding domain plays a role in Nrg1-Rtg3 binding to the *ALT2* promoter, chromatin immunoprecipitation experiments were carried out. Nrg1^-^*^Myc13^* binding increased threefold in the promoter region of *ALT2* bearing the Nrg1 consensus binding sequence (TCCC), as compared with the rest of the promoter (figure 3*a*). An *RTG3* mutant tagged with Myc13 was constructed as stated in Methods, in order to compare Nrg1^-*Myc13*^ Rtg3 versus Rtg3^-*Myc13*^ Nrg1 binding to the *ALT2* promoter. As shown in figure 3*a*, as well as Nrg1^-*Myc13*^ Rtg3 binding, that of Rtg3^-*Myc13*^ Nrg1 was increased fourfold in the same region, suggesting that the two activators act together in a complex, Nrg1 recruiting Rtg3 to the Nrg1 DNA-binding domain. Consequently, chromatin immunoprecipitation was performed in *RTG3^-Myc13^ NRG1, RTG3^-Myc13^ nrg1*Δ, *RTG3 NRG1^-Myc13^* and *rtg3*Δ *NRG1^-Myc13^* mutant strains. Figure 3*b,c* shows that Nrg1 binding is Rtg3 independent, while Rtg3 does not bind to the *ALT2* promoter in an *nrg1*Δ mutant background. Thus, Rtg3 promoter binding depends on Nrg1.

**Figure 4.**
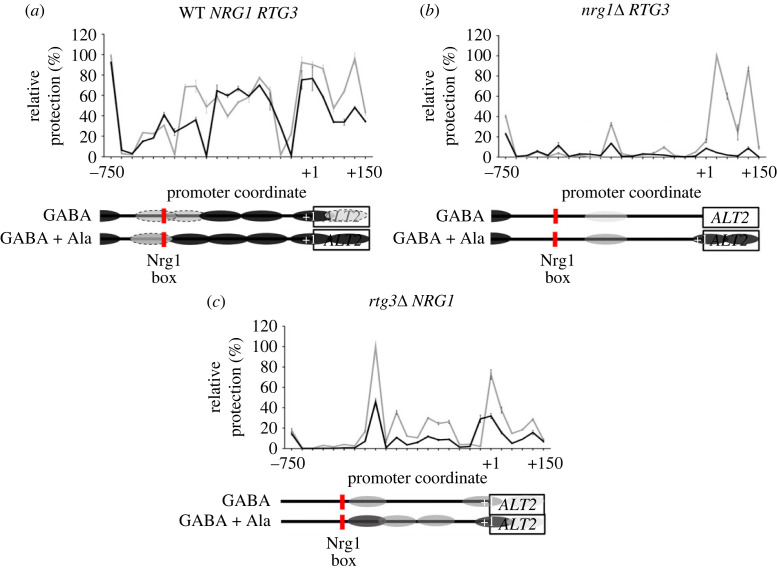
Both, Nrg1 and Rtg3 are necessary for chromatin rearrangement in the *ALT2* promoter. Nucleosome scanning assay (NuSA) samples were obtained as follows: wild type (WT) or mutant strains were grown on MM glucose (2%) + GABA (7 mM) or glucose (2%) GABA (7 mM) + alanine (7 mM). When each culture reached an OD_600nm_ = 0.6, samples of both cultures were taken for nucleosome scanning assay, as described in Methods. Mean values of three independent experiments are shown. Black lines show chromatin organization observed on GABA and grey lines on GABA + ala grown cells. In the *ALT2* depicted promoter, vertical red line shows Nrg1 consensus binding sequence, black ovals over x-axis indicate firmly positioned nucleosomes and dotted ovals depict fuzzy nucleosomes. (*a*) WT, (*b*) *nrg1*Δ and (*c*) *rtg3*Δ.

Analysis of *ALT2* chromatin organization showed that the *ALT2* promoter was condensed when the wild-type strain was grown with GABA as the sole nitrogen source (figure 4*a*). When GABA and alanine were simultaneously provided, *ALT2* promoter organization was further compacted (figure 4*a*), in line with the repressive role of alanine. In both *nrg1*Δ and *rtg3*Δ single mutants, *ALT2* promoter chromatin organization was relaxed, in accordance with the *ALT2* de-repressed expression seen in these strains (figure 4*b,c*, and figure 1*c*).

### 3.3. Absence of either Nrg1 or Rtg3 results in loss of respiratory metabolism and mitochondrial DNA

**Figure 5.**
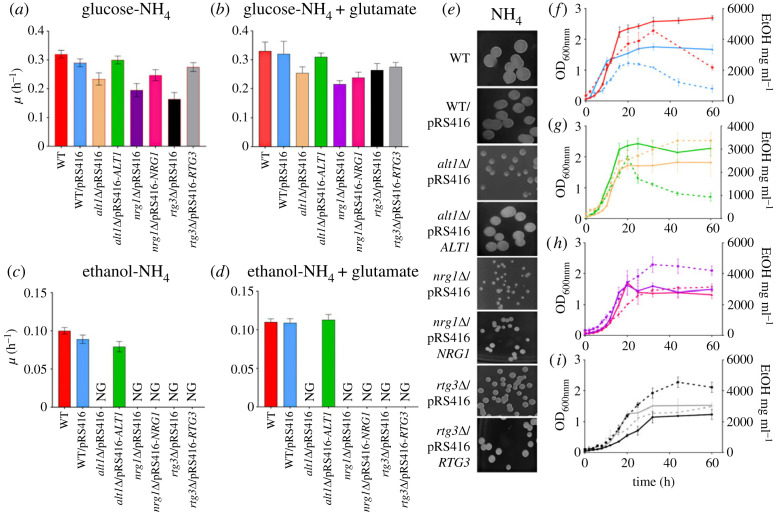
*nrg1*Δ and *rtg3*Δ mutants show a petite phenotype even when transformed with their cognate *NRG1* or *RTG3* wild-type genes. (*a*) Specific growth rate of MM glucose (2%)-ammonium (40 mM); (*b*) specific growth rate on MM glucose (2%)-ammonium (40 mM) + glutamate (5 mM); (*c*) specific growth rate in MM ethanol (2%)-ammonium (40 mM); (*d*) specific growth rate on ethanol-ammonium + glutamate (5 mM). The results of three independent experiments are presented. (*e*) Colony size in glucose + ammonium medium. Plates were incubated at 30°C for 3 days on glucose ammonium (NH_4_). (*f*–*i*) Cells were cultured on MM glucose + ammonium to monitor biomass (solid lines) and ethanol concentration (dotted lines). Each line represents the average of three independent experiments.

It has been previously reported that *rtg3*Δ and not *nrg1*Δ mutants are glutamate bradytrophs, since Rtg3 absence results in the loss of the retrograde response, hindering the transcriptional activation of *CIT1*, *ACO1* and *IDH1/2*, necessary for glutamate biosynthesis [21]. Figure 5*a,b* shows that, as expected, *rtg3*Δ mutants are glutamate bradytrophs, which when grown in the presence of glutamate or transformed with pRS416-*RTG3* strains regain glutamate prototrophy, confirming the recovery of the *RTG3* wild-type phenotype.

The presence of the transforming plasmid was monitored by complementation of uracil auxotrophy, which is present in the receiving mutant strains. As detailed in the Methods section, *nrg1*Δ and *rtg3*Δ mutants were obtained by gene replacement, transforming wild-type strains with *kanMX* modules directing their insertion into the *NRG1* or *RTG3* coding sequences, respectively, to obtain the cognate *nrg1*Δ or *rtg3*Δ null mutants. Ten kanamycin-resistant mutants were selected from each transformation, purified and analysed by PCR, confirming the correct insertion of the *kanMX* module. None of the transformed kanamycine-resistant strains were able to grow on ethanol as the sole carbon source and all showed a petite phenotype, forming small colonies when grown on glucose as a carbon source. This observation was confirmed by two independent transformation experiments for each of the two inactivated genes (figure 5*e*).

Deletion of *NRG2* (*nrg2*Δ), which is the paralog of *NRG1* in *S. cerevisiae* engineered with an identical methodology, did not result in a petite phenotype nor did it impair growth on ethanol as a carbon source. The clear phenotype of *nrg1*Δ mutants implies that Nrg2 cannot fulfil the Nrg1 physiological function. However, we constructed a *nrg2*Δ single mutant and a *nrg1*Δ *nrg2*Δ double mutant, as described in Methods. As expected, a *nrg2*Δ mutant grew on both ethanol and glucose as carbon sources, showing a wild-type phenotype, whereas the double mutant *nrg1*Δ *nrg2*Δ showed a petite phenotype on glucose and did not grow on ethanol as the sole carbon source (electronic supplementary material, figure S3).

To further confirm that Nrg1 or Rtg3 lack resulted in a petite phenotype, *nrg1*Δ and *rtg3*Δ strains were also obtained from the BY4742 strain collection (Open Biosystems) (table 1). The growth phenotype of these mutants was analysed, and as electronic supplementary material, figure S4 shows, BY4742 *nrg1*Δ and BY4742 *rtg3*Δ did not grow on ethanol and showed decreased growth rate when grown on glucose as sole carbon source, as compared with the wild-type strain.

**Figure 6.**
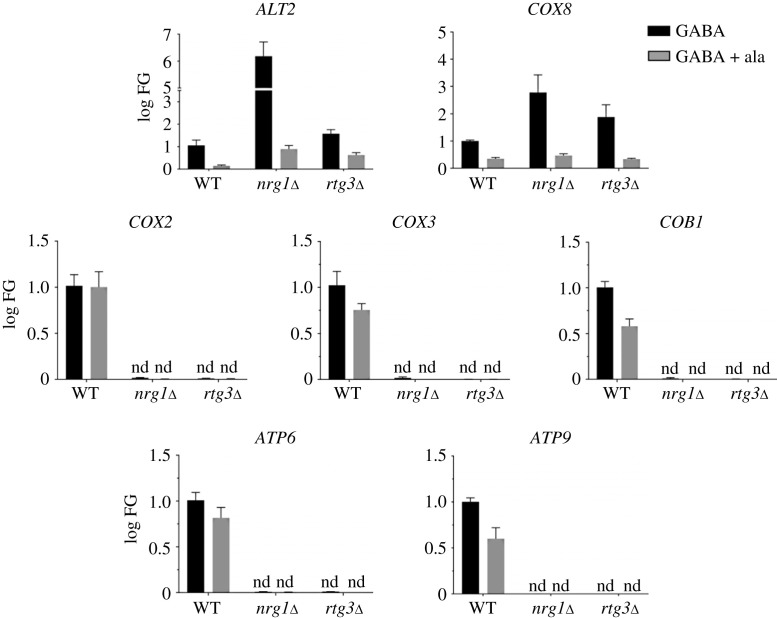
Nrg1-Rtg3 complex is necessary for mitochondrial DNA expression. Strains were grown on MM glucose (2%) as carbon source and GABA (7 mM) as nitrogen source (black bars), or MM glucose (2%) as carbon source and GABA (7 mM) + alanine (7 mM) as nitrogen sources (grey bars), and collected at OD_600nm_ = 0.3. Gene expression was measured by qPCR using 18S as constitutive control by the 2^^-*Δ*ΔCT^ method. qPCR analysis shows *ALT2* and *COX8* nuclear genes expression in the *NRG1 RTG3*, *nrg1*Δ *RTG3* and *NRG1 rtg3*Δ strains, grown on GABA (7 mM) or GABA (7 mM) + alanine (7 mM), indicating these are repressed in the wild-type strain and de-repressed in *nrg1*Δ mutant. The *COX2*, *COX3*, *COB1*, *ATP9* and *ATP6* mitochondrial genes expression is absent in *rtg3*Δ and *nrg1*Δ mutants. Black bars, MM glucose and GABA; grey bars, MM glucose and GABA + alanine; nd, not detected.

Analysis of the ability to generate and consume ethanol by the *nrg1*Δ and *rtg3*Δ mutants was analysed, showing that *nrg1*Δ and *rtg3*Δ mutant strains could produce ethanol but were unable to consume it (figure 5*h,i*). The above results indicate that the absence of either *NRG1* or *RTG3* results in a petite phenotype. Moreover, when we attempted to complement the *nrg1*Δ and *rtg3*Δ strains with the cognate wild-type genes, the ability to grow on ethanol was not recovered and neither was the petite phenotype reversed (figure 5*c,d*). These results confirmed that the absence of either Nrg1 or Rtg3 results in irreversible damage to the mitochondrial function (figure 5*f,i*). Furthermore, the transformed strains *rtg3*Δ/pRS416-*RTG3* and *nrg1*Δ/pRS416-*NRG1* did not recover ethanol consumption capacity (figure 5*h,i*). As a control, ethanol production and consumption were analysed in the petite strain *alt1*Δ [19] and in the cognate strain transformed with pRS416-*ALT1* (figure 5*g*). In this case, *alt1*Δ harbouring the empty plasmid pRS416 produced but did not consume ethanol, whereas a strain transformed with pRS416-*ALT1* recovered the wild-type capacity to produce and consume ethanol and did not show a petite phenotype (figure 5*e,g*). To further analyse the roles of Rtg3 and Nrg1 in the regulation of mitochondrially encoded genes, we determined the expression of five mitochondrial DNA-encoded genes. *Saccharomyces cerevisiae* mitochondrial DNA encodes cytochrome oxidase subunits I, II and III (*COX1, COX2*, *COX3*), apocytochrome b (*COB1*), and three ATP synthase subunits (*ATP6, ATP8*, *ATP9*) [48–51]. Since mitochondrial genes are expressed as polycistronic transcripts, we determined the expression of five of the above-mentioned genes (*COX2, COX3*, *COB1*, *ATP6* and *ATP9*) representing different transcriptional units. As shown in figure 6, expression of these genes was observed in the wild-type strain and was completely absent in either the *nrg1*Δ or *rtg3*Δ mutants grown on glucose-GABA or glucose-GABA + alanine. These results confirm that the absence of either partner of the chimeric regulator results in irreversible damage to the mitochondria, correlated with the loss of mitochondrial DNA. We further analysed the expression of *ALT2* and the nuclear gene *COX8*, which encodes cytochrome c oxidase subunit VIII [34] and *ALT2* genes. As shown in figure 6, the expression of *ALT2* is repressed in the wild-type strain when grown on either GABA or GABA + alanine. Under both growth conditions, *ALT2* expression is de-repressed in *nrg1*Δ and *rtg3*Δ strains. However, *COX8* is not de-repressed on GABA alanine in either mutant, indicating that *COX8* regulation does not entirely depend on the Nrg1/Rtg3 chimeric protein. On GABA the alanine pool is lower than on GABA alanine, thus de-repression can occur more readily for both *ALT2* and *COX8.* Chromatin of the *COX8* promoter is presumably condensed in the presence of alanine as it is shown for *ALT2* in figure 4.

**Figure 7.**
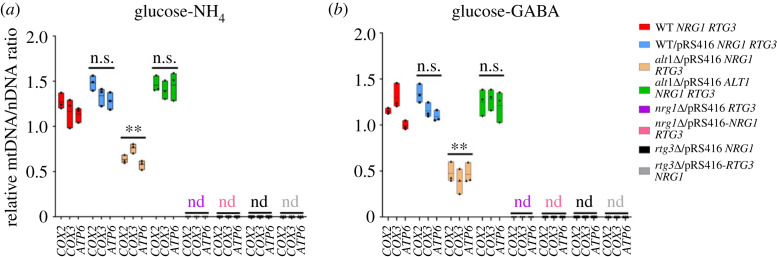
Both *nrg1*Δ and *rtg3*Δ result in drastic depletion of mitochondrial DNA. Cells were harvested on (*a*) MM glucose (2%) + ammonium (NH_4_, 40 mM) or (*b*) MM glucose (2%) + GABA (7 mM) and collected at OD_600nm_ = 0.6. Estimation of mtDNA/nDNA ratio by qPCR using *COX2, COX3* and *ATP6* as mitochondrial genes; this determination is relative to the *COX8* encoded nuclear gene. Boxes show individual values (points) and the average (middle line) + s.d. of three independent experiments. Statistical analysis was done using two-way ANOVA. Asterisks indicate a significant difference ** = *p* < 0.005. n.s., non-significant; nd, not detected.

We determined the mtDNA/nDNA ratio by qPCR using *COX8* as a nuclear control. DNA was quantified in samples obtained from glucose-GABA- or glucose-ammonium-grown cells, mtDNA was completely absent in the *nrg1*Δ and *rtg3*Δ deleted strains as shown by the mtDNA/nDNA ratio (figure 7*a,b*), regardless of whether they were re-transformed with cognate *NRG1* or *RTG3* wild-type genes. As expected, identically to the CLA1-2-derived mutants, BY4742 *nrg1*Δ and *rtg3*Δ strains displayed decreased mitochondrial DNA, showing a very low rate of mtDNA versus nDNA (electronic supplementary material, figure S5).

As a control, we measured mitochondrial DNA in *alt1*Δ strains. In these strains, the total amount of mitochondrial DNA is reduced by 70%; however, and differently to what we observe, that in strains deleted for either *NRG1* or *RTG3*, mitDNA is fully recovered upon complementation with the *ALT1* wild-type gene [19] (figure 7*a,b*).

**Figure 8.**
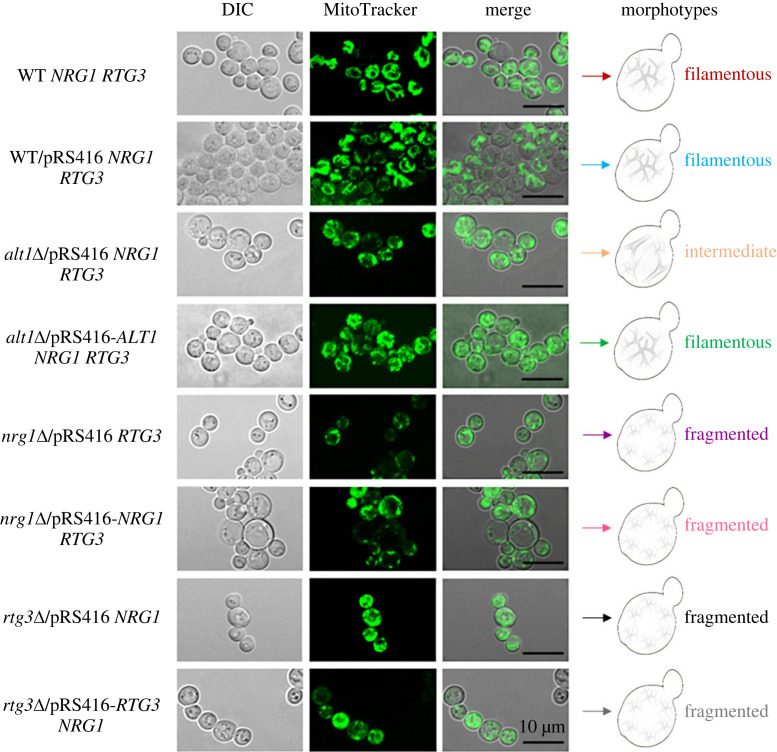
*nrg1*Δ and *rtg3*Δ show a drastically altered mitochondrial morphology. Mitochondrial staining with MitoTracker Green FM 9074 (Molecular Probes) was used to determine the morphology of the mitochondria in each strain. The black scale-bar corresponds to 10 µm. Morphotypes show a schematic representation of the mitochondrial organization in different strains.

Thus, both Nrg1 and Rgt3, acting in a complex, play a crucial role in maintaining mitochondrial DNA integrity and/or stability. Consequently, we examined the appearance of mitochondria, as monitored by the MitoTracker Green. Figure 8 shows that the appearance of mitochondria in both the wild type and *alt1*Δ strains complemented with *ALT1* showed a typical filamentous organization, while *alt1*Δ presented a clearly diminished filamentous organization [19]. This contrasts with the fragmented pattern observed in both the *nrg1*Δ and *rgt3*Δ strains, whether complemented or not with their cognate wild-type genes (figure 8).

**Figure 9.**
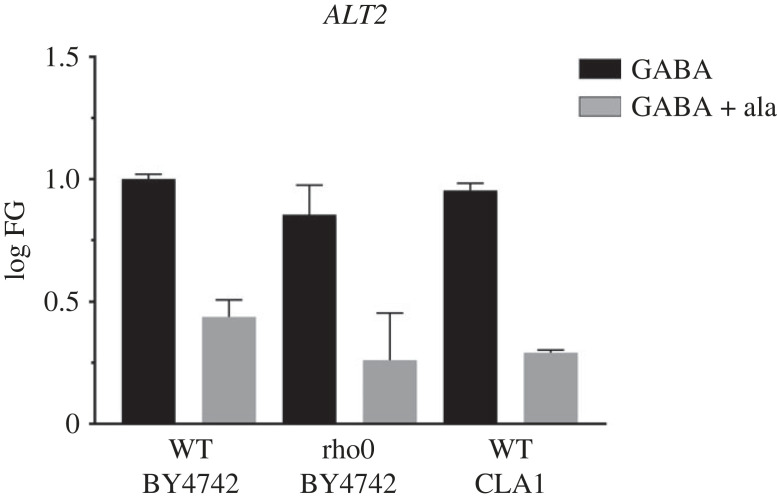
*ALT2* alanine-dependent repression is observed in the BY4742 rho0 strain. Cells were collected at OD_600nm_ = 0.3 on MM glucose 2% GABA 7 mM) (black bars) and MM glucose 2% GABA 7 mM + alanine 7 mM MM glucose (grey bars). Gene expression was measured by qPCR using 18S as constitutive control by the 2^^−*Δ*ΔCT^ method. qPCR analysis shows *ALT2* nuclear genes expression in the WT BY4742, BY4742 rho0 and WT CLA1-2.

To further analyse the role of the chimeric Nrg1-Rtg3 as a repressor, we determined *ALT2* expression in GABA and GABA + alanine in a BY4742 derivative rho0 mutant strain, unable to grow on ethanol as sole carbon source (electronic supplementary material, figure S5), whose NRG1/RTG3 wild-type phenotype was confirmed by qPCR. As figure 9 shows, *ALT2* expression was alanine repressed, showing that the alanine-mediated regulation of at least *ALT2* in the *nrg1*Δ and *rgt3*Δ strains is not a secondary consequence of the loss of mitochondrial DNA.

### 3.4. Modelling of the Nrg1/Rtg3 complex, the unicity of Nrg1

**Figure 10.**
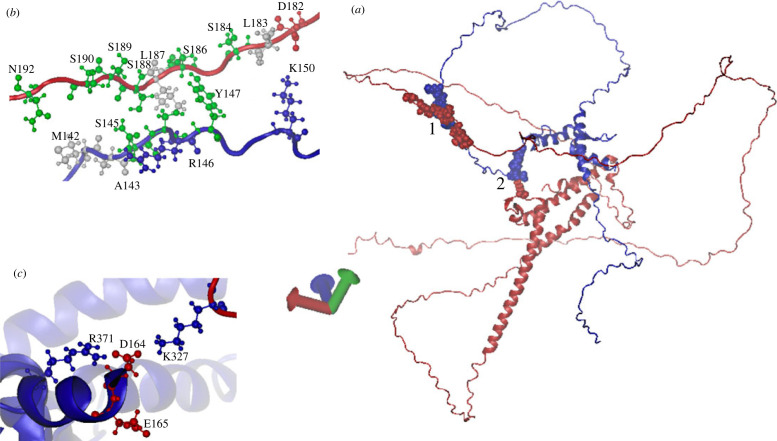
Modelling the Nrg1/Rtg3 interaction. Panel (*a*), most likely model of the Nrg1/Rtg3 chimaeric molecule obtained by ColabFold v. 1.5.2: AlphaFold2 using MMseq (https://colab.research.google.com/github/sokrypton/ColabFold/blob/main/AlphaFold2.ipynb#scrollTo=ADDuaolKmj) [42]. In all panels, blue chain, Nrg1; red Chain, Rtg3. The residues in the two contact patches labelled 1 and 2 are shown for clarity as van der Waals (VDW; space-filling). Panels (*b,c*), individual residues involved in the two contact patches shown in panel (*a*), as follows: panel (*b*), individual residues contacting the two extended intrinsically disordered regions labelled 1 in panel (*a*). Panel (*c*), individual residues in the patch labelled 2 in panel (*a*), highlighting the contacts of Lys327 of Rgt3 with acidic residues of Nrg1. Contacts between residues as determined with the MAPIYA contact server; (https://mapiya.lcbio.pl/, MAPIYA contact map server for identification and visualization of molecular interactions in proteins and biological complexes [43]).

We attempted to investigate the structure of the Nrg1/Rtg3 complex through its modelling using the AlphaFold2 database (see Methods). Figure 10 shows the most probable complex deduced. Interestingly, neither of the components of the complex can be superimposed on the AlphFold2 models (available in the database *NRG1* AF-A0A815XE22-F1-model_ v4.pdb and *RTG3* AF-A0A815WEC9-FI-model_ v4.pdb) through the Multiseq plugin available in VMD [52], which may suggest, with the provision of the low confidence of the AlphFold2 result, it is tentative to consider that the formation of the complex alters the three-dimensional configuration of both components. We analysed the possible contacts between the components of the complex [53]. They lie in two patches. A number of contacts exist between the two intrinsically disordered stretches (see figure 10 legend). An additional contact was made between Lys327 of Rtg3 and Asp164 and Glu165 of Nrg1 (figure 10, see also electronic supplementary material, figure S1). These contacts provide a clear rationale for the unique specificity of Nrg1. Nrg1 and Nrg2 show a global 50.46% identity; however, our results show clearly that Nrg2 cannot substitute Nrg1 as a component of the chimeric regulator, as it suffices to delete Nrg1 to acquire the respiratory defective petite phenotype (see below). Nrg1 neo-functionalization resulted in the acquisition of pertinent amino acid residues that could support Nrg1–Rtg3 interactions. Nrg1 and Nrg2 differed between residues 142 (Met in Nrg1) and 171 (Arg in Nrg1) where the main patch of contact residues was located. Moreover, Asp164 and Glu165, which form a salt bridge and H bonds with Lys327 of Rtg3, are absent in Nrg2. The predicted AlphFold2 models for Nrg1 and Nrg2 were also substantially different (electronic supplementary material, figures S1 and S2).

## 4. Discussion

### 4.1. Alanine as a metabolic signal promoting organization of the Nrg1–Rtg3 modulator

Alanine aminotransferases catalyse reversible transamination between alanine and *α*-ketoglutarate, leading to pyruvate and glutamate. At least two alanine aminotransferase isozymes, generated through independent gene duplication events, occur frequently in animals, plants, yeasts and bacteria, and are proposed to be involved in the main pathways of both alanine biosynthesis and catabolism [15,54–58]. Amino acid metabolism has been thoroughly studied in *S. cerevisiae*; however, no alanine auxotrophs have been isolated from this yeast. Moreover, although Alt1 is the main alanine biosynthetic enzyme contributing 80% of the alanine intracellular pool, *alt1*Δ mutation does not lead to alanine auxotrophy, indicating that at least one additional pathway for alanine biosynthesis must be present in this yeast [17,59]. There are two candidates for an alternative alanine biosynthesis pathway: the glutamine-pyruvate aminotransferase [59–62], and the *UGA1*-encoded GABA transaminase, where the resulting succinate semialdehyde is readily oxidized through Uga2, rendering alanine biosynthesis irreversible [19]. Thus, it is likely that in *S. cerevisiae* an alanine intracellular pool is permanently available [19], which could facilitate the assembly of the chimeric Nrg1-Rtg3 regulator described here.

Alanine acts as an *ALT1* positive regulator inducer and an *ALT2* co-repressor, while also repressing the expression of genes involved in the GABA shunt [19]. As the formation of the Nrg1-Rtg3 complex is enhanced in the presence of alanine, this suggests that this amino acid may play an additional regulatory role in facilitating and/or promoting the assembly of this chimeric regulator. Our results showed that even with proline as the sole nitrogen source, a condition in which the alanine intracellular pool is reduced [19], the Nrg1–Rtg3 chimeric complex is formed (figure 2*c*). Additionally, we showed that *ALT2* Nrg1-Rtg3-dependent repression was observed even in cultures grown on GABA in the absence of alanine (figure 6), further supporting that Nrg1–Rtg3 assembly could be mediated by the intracellular alanine pool.

### 4.2. Nrg1-Rtg3-dependent regulation is essential to maintain DNA integrity and mitochondrial function

We demonstrated that Nrg1 and Rtg3 are chimeric transcriptional regulators. Analysis of the homologous pair, Nrg1 and Nrg2, showed a global identity of 50.46%. However, *NRG2* cannot substitute for the *NRG1*-encoded component of the chimeric regulator, as *nrg2*Δ mutants do not show the *nrg1*Δ or *rtg3*Δ mutant phenotypes. Indeed, neo-functionalized Nrg1 acquired amino acid residues that can be predicted to mediate Nrg1–Rtg3 interaction, which is absent in Nrg2. When the Nrg1–Rtg3 chimera cannot be assembled, as in the *nrg1*Δ or *rtg3*Δ mutants, mitochondrial DNA is irreversibly damaged, resulting in extreme mitochondrial fragmentation (figure 8), thus cells cannot carry out respiratory metabolism. When these mutants are transformed with the *NRG1* or *RTG3* wild-type genes, the capacity to carry out respiratory metabolism is not recovered, since mitochondrial DNA has been irreversibly lost. The fact that both *nrg1*Δ and *rtg3*Δ single mutants result in loss of mitochondrial DNA strongly suggests that this phenotype results from the absence of the Nrg1–Rtg3 chimeric complex. This is a hitherto undescribed function that is not carried out by individual, un-complexed Nrg1 or Rtg3 regulators. It is unlikely that the new role of the Nrg1–Rtg3 chimeric complex in maintaining mitochondrial DNA integrity could be related to its role in repressing *ALT2*, considering that *alt2*Δ mutants do not show any obvious phenotype. We favour the idea that the Nrg1-Rtg3 complex plays additional regulatory roles, positive or negative, on a gene or genes crucially involved in mitochondrial DNA replication and/or maintenance.

Different high-throughput studies aimed at detecting nuclear genes affecting mitochondrial function have failed to obtain *nrg1* or *rgt3* defective mutants [63] which seems surprising, given the results described in this paper. This apparent discrepancy may be due to different genetic backgrounds from the one we used. However, in four of the reported studies, BY4742 or its derivatives were used. This is one of the strains from which we derived *nrg1*Δ and *rtg3*Δ showing a petite phenotype, so it is more likely that in the studies reported in Stenger *et al.* [63] the class of nuclear genes affecting mitochondrial function was saturated. This appears clearly from the fact that only 113 deletions, out of the approximately 300 deletions, detected in each study, were found in all the four studies reviewed. Thus *nrg1*Δ and *rgt3*Δ strains could have been missed.

The Nrg1-Rtg3 chimeric regulator is the result of the interaction of these two regulatory proteins, which acquired the possibility to interact through neo-functionalization, confirming the importance of the existence of multiple cooperative protein–protein interactions (29). The formation of chimeric complexes such as Hap2-3-5-Gln3 [27], Mat*a*2, Mcm1 and Nrg1-Rtg3 supports the notion that extant elements can be recruited to novel functions through their interactions leading to chimeric regulators [64,65].

In addition to its traditional mitochondrial role in energy transformation, this organelle is involved in many other crucial functions. Mitochondria are vital for sustaining cellular and organismal pathways that determine the direction of metabolism and develop pertinent stress responses and cellular fate [66]. To fully accomplish these tasks, intracellular communication networks depend on diverse molecular cascades. Indeed, feedback from mitochondria is required to coordinate mitochondrial biogenesis and/or removal by mitophagy during the division cycle [66], and a coordinated regulation of these processes by transcription factors, such as the Nrg1–Rtg3 chimeric complex, can allow cellular adaptations to stress and other environmental modifications [66,67].

When glucose becomes limited, *S. cerevisiae* cells undergo a diauxic shift, resulting in respiratory metabolism. Under these conditions, *S. cerevisiae* relies on aerobic energy production to support growth. The absence of either component of the chimeric Nrg1–Rtg3 regulator results in the loss of respiratory metabolism, leading to a fully fermentative metabolism. Such facultative properties of *S. cerevisiae* allowed us to uncover a novel system involved in the maintenance of mitochondrial DNA integrity. It would be interesting to determine if this same system is present in obligatory aerobic yeasts and the evolution of analogous systems in other eukaryotes.

## 5. Conclusion

**Figure 11.**
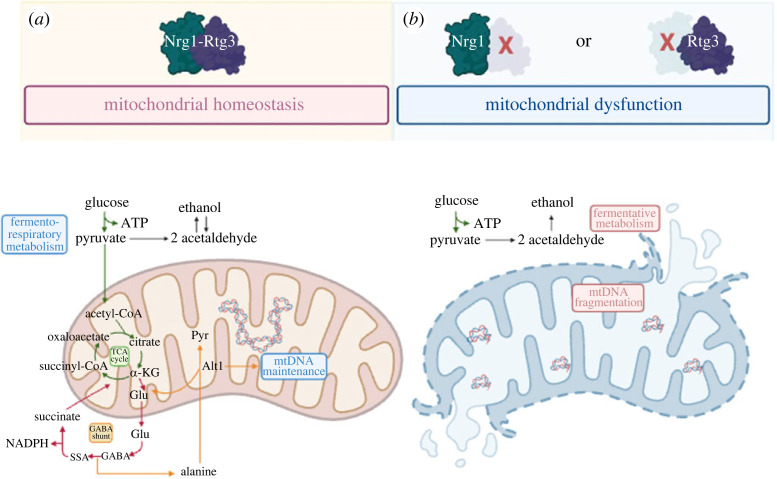
Nrg1–Rtg3 chimeric transcriptional modulator is essential to maintain mitochondrial DNA integrity in *S. cerevisiae*. After neo-functionalization, the Nrg1 regulator gained amino acid residues, which allowed the assembly of the Nrg1–Rtg3 novel chimeric modulator, which plays an important role in the maintenance of mitochondrial DNA (mtDNA) integrity due to an unknown mechanism (panel (*a*)). The mitochondrial function produces important molecules, the metabolism of which plays a key role in stress protection. Our results indicate that in the absence of Nrg1 (*nrg1*Δ) or Rtg3 (*rtg3*Δ), the chimeric Nrg1–Rtg3 regulator is not assembled, causing mitochondrial dysfunction, which results in a lack of mtDNA and null respiratory metabolism, which affects essential mitochondrial functions such as the TCA cycle, stress response, apoptosis, mitophagy, and ageing (panel (*b*)). Succinic semialdehyde (SSA), α-ketoglutarate (α-KG), glutamate (Glu) and pyruvate (Pyr). Image created using BioRender.

Metabolic adaptation to diverse and new environments results in complex modifications of cell physiology, allowing them to activate relevant response pathways. Although all organisms include a vast array of DNA-binding proteins participating in adaptive transcriptional responses, the formation of chimeric modulators, combining different DNA-binding and activation domains, can expand the repertoire of activators or repressors, allowing the development of novel and more complex responses [26,27]. This is the case for the Nrg1–Rtg3 chimeric modulator described here as essential for maintaining respiratory metabolism and mitochondrial-dependent energy production (figure 11).

## Data Availability

The datasets supporting this article have been uploaded as part of the electronic supplementary material [68].
